# Monoclonal Antibodies and Antibody Drug Conjugates in Multiple Myeloma

**DOI:** 10.3390/cancers13071571

**Published:** 2021-03-29

**Authors:** Jakub Radocha, Niels W. C. J. van de Donk, Katja Weisel

**Affiliations:** 1 4th Department of Internal Medicine-Hematology, Faculty of Medicine in Hradec Králové, University Hospital Hradec Kralove, Charles University, 50005 Hradec Kralove, Czech Republic; 2 Department of Hematology, Cancer Center Amsterdam, Amsterdam UMC, Vrije Universiteit Amsterdam, 1081 HV Amsterdam, The Netherlands; n.vandedonk@amsterdamumc.nl; 3 II Medizinische Klinik und Poliklinik, Universitätsklinikum Hamburg-Eppendorf, 20251 Hamburg, Germany; k.weisel@uke.de

**Keywords:** multiple myeloma, monoclonal antibodies, antibody drug conjugates

## Abstract

**Simple Summary:**

Monoclonal antibodies represent a major therapeutic progress in multiple myeloma during the last decade. The use of antibodies as well as antibody drug conjugates has changed the treatment landscape rapidly. The intent of this paper is to summarize the current major results of monoclonal antibody treatments in multiple myeloma.

**Abstract:**

Multiple myeloma is the second most common hematologic malignancy. Current treatment strategies are mainly based on immunomodulatory drugs, proteasome inhibitors or combination of both. Novel agents added to these backbone treatments represent a promising strategy in treatment of newly diagnosed as well as relapsed and refractory multiple myeloma patients. In this respect, the incorporation of monoclonal antibodies into standard-of-care regimens markedly improved prognosis of myeloma patients during the last years. More specifically, monoclonal anti-CD38 antibodies, daratumumab and isatuximab, have been implemented into treatment strategies from first-line treatment to refractory disease. In addition, the monoclonal anti-SLAM-F7 antibody elotuzumab in combination with immunomodulatory drugs has improved the clinical outcomes of patients with relapsed/refractory disease. Belantamab mafodotin is the first approved antibody drug conjugate directed against B cell maturation antigen and is currently used as a monotherapy for patients with advanced disease. This review focuses on clinical efficacy and safety of monoclonal antibodies as well as antibody drug conjugates in multiple myeloma.

## 1. Introduction

Multiple myeloma (MM) is a clinically heterogeneous disease, as evidenced by considerable variation in rates of response to treatment and overall survival (OS); indeed, OS in patients with MM has been shown to range from a few months to more than a decade [1]. MM has long represented a therapeutic challenge. After introduction of proteasome inhibitors and immunomodulating agents, the landscape of treatment is still rapidly evolving, and monoclonal antibodies (MoAb) have become an integral part of the myeloma therapeutic approach. Myeloma cells carry several potential targets for immunotherapy with CD38 and B cell maturation antigen (BCMA) being the most widely studied. In this review, we will focus on basic mechanisms of action, and especially on the clinical efficacy and safety of various monoclonal antibodies used in MM treatment.

## 2. CD38

CD38 is a transmembrane glycoprotein first described more than 40 years ago in 1980 as a marker of T cell differentiation [2]. The molecule is expressed and distributed not only on plasma cells but also on other myeloid and lymphoid cells [3]. High expression of CD38 can be observed on natural killer (NK) cells and subsets of T lymphocytes [4]. Other immune effector cells also show high expression of CD38 including regulatory B cells and antigen presenting cells (APC), especially plasmacytoid dendritic cells. A decrease in plasmacytoid densdritic cells, which support MM cell growth and survival, may represent another potent immune effect of CD38 antibodies [5,6]. CD38 works as an enzyme (ectoenzyme) at it also can serve as a receptor triggering proliferation signals [7]. As an enzyme, it is involved in the catabolism of nicotinamide adenine dinucleotide (NAD+) and nicotinamide adenine dinucleotide phosphate (NADP). Some studies suggest that CD38 is involved in the production of adenosine. MM cells grow in an environment rich with adenosine and levels of adenosine are higher in the bone marrow of MM patients compared to patients with monoclonal gammopathy of undetermined significance [8]. It was recently reported that so called mitochondrial transfer from stromal plasma cells to malignant MM cells via the tumor-derived tunneling nanotubes is facilitated by CD38 molecules which leads to enhancement of MM cells energy sources [9]. Other functions of CD38 were demonstrated in CD38 knockout mouse models. For example, loss of CD38 makes mice susceptible to bacterial infections due to impaired neutrophil migration [10]. In addition, CD38 regulates the migration of dendritic cell precursors from the blood to peripheral sites [11].

## 3. Anti-CD38 Monoclonal Antibodies

Several MoAbs targeting CD38 are currently available for MM treatment either as approved drugs (daratumumab and isatuximab) or still in clinical development (MOR202 and TAK-079). Daratumumab, a fully human IgG1-k antibody, was the first approved antibody for the treatment of MM patients, briefly followed by isatuximab (chimeric IgG1-k antibody), and MOR202, fully human IgG1-l antibody was studied in phase I/II clinical trial but to date, no other trials are ongoing in MM [12]. TAK-079 (fully human monoclonal antibody) binds to CD38 with high affinity and currently two clinical trials in MM are ongoing) [13].

### 3.1. Mechanism of Action of CD38 Monoclonal Antibodies

**Figure 1:**
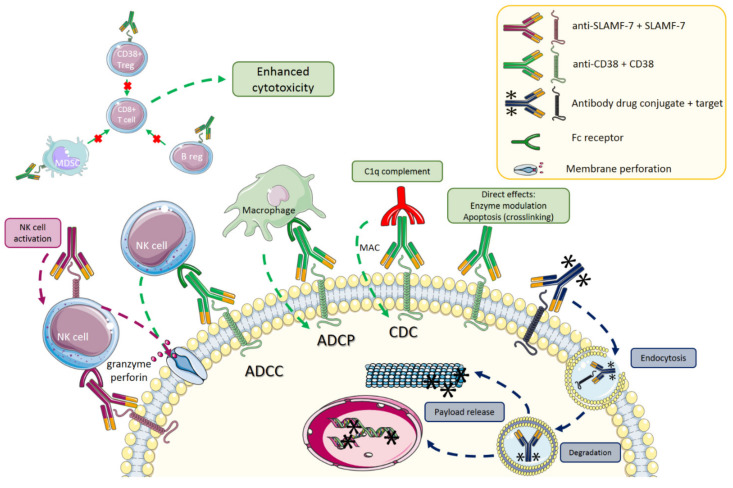
Mechanism of action of different naked antibodies and antibody drug conjugates. Effects of SLAMF-7 in purple, CD38 in green, and antibody drugconjugates (ADC) dark blue. Abbreviations: MDSC: myeloid-derived suppressor cells, NK: natural killer, Treg: regulatory T cell, B reg: regulatory B cell, ADCC: antibody dependent cellular cytotoxicity, ADCP: antibody dependent cellular phagocytosis, CDC: complement dependent cytotoxicity, MAC: membrane attacking complex. Images used from Servier Medical Art (available at https://smart.servier.com/, accessed date 20 February 2021, licensed under a Creative Commons Attribution 3.0 Unported License (CC BY 3.0, https://creativecommons.org/licenses/by/3.0/ accessed date 20 February 2021).

All described antibodies show Fc-dependent mechanisms of action [14]. Antibody-dependent cellular cytotoxicity, as well as complement dependent cytotoxicity are prolific mechanisms of action in anti-CD38 treatment [15]. In addition, all molecules display antibody-dependent cellular phagocytosis as one of the modes of action [16]. Direct proapoptotic effect has also been described for isatuximab [17]. Since these CD38-targeting antibodies target different CD38 epitopes, the mechanisms of action slightly differ among the drugs. Daratumumab has the strongest complement-dependent cytotoxicity (CDC) and antibody-dependent cellular phagocytosis (ADCP) activity whereas isatuximab has the strongest direct proapoptotic effect and is capable of inducing cell death without crosslinking [18]. Isatuximab is also capable of modulating the enzymatic function of CD38 [19]. Based on preclinical data, blocking CD38 prevents mitochondrial trafficking from stromal cells to MM cells thereby possibly depleting energy sources for MM cell growth [9,20]. Since CD38 is expressed on other immune cells, other mechanisms of action include immunomodulatory effects especially seen on T and NK cells [4]. The observed decrease in Treg numbers could enhance NK and T cell mediated antitumor response as shown in experimental models with daratumumab and isatuximab [4,19]. Daratumumab reduces CD38 expression on normal cells as well [21]. Increase in granzyme B activity in T cells was also documented with daratumumab [22]. The most important mechanisms of action of MoAbs discussed in this review are shown in Figure 1.

### 3.2. Clinical Efficacy of Anti-CD38 Monotherapy

Efficacy and safety of daratumumab monotherapy was demonstrated in the two pivotal trials in patients with relapsed/refractory MM (RRMM): GEN501 and SIRIUS trials [23,24]. Based on the results of these trials, daratumumab has been approved by Food and Drug Administration (FDA) in 2015 and by European Medicines Agency (EMA) in 2016. Briefly, the overall response rate with daratumumab monotherapy in pooled analysis was 31.1% with a median progression free survival of 4.0 months [25]. Both trials included heavily pretreated patients with median number of five previous lines. The updated final analysis confirmed existence of durable responses in heavily pretreated patients after daratumumab monotherapy [26]. The usual dosage of daratumumab (as well as other MoAbs) is summarized in Table 1.

Isatuximab monotherapy was evaluated in phase I/II trial (NCT01084252) where patients received isatuximab as monotherapy or in combination with dexamethasone [27]. Patients received median of four prior lines of therapy. In total, 72% of patients were double refractory to proteasome inhibitor (PI) and immunomodulatory drug (IMiD) and 7% were quadruple refractory. Overall response rate (ORR) was 23.9% with isatuximab monotherapy arm and 43.6% with the combination of isatuximab plus dexamethasone. Median progression free survival (PFS) was 4.9 months with isatuximab monotherapy arm and 18.9 months with isatuximab plus dexamethasone. No unexpected safety issues were detected. 

The first study evaluating MOR202 was a phase 1/2a trial (NCT01421186) with either MOR202 monotherapy or MOR202 in combination with dexamethasone, lenalidomide or pomalidomide. The trial included patients with median 2–4.5 prior treatment lines depending on the arm of the study. No responses were observed with MOR202 monotherapy at doses between 0.01 and 16 mg/kg. Responses were seen only in subsequent combinations with dexamethasone (28%), lenalidomide (65%) and pomalidomide (48%). No unexpected safety issues were seen [12]. Currently there are no other ongoing trials with MOR202. TAK-079 as single agent was associated with an ORR of 43% in heavily pretreated MM patients in the first-in-human phase Ib trial (28 patients were treated, 94% of patients were refractory to a PI or IMiD) [28].

### 3.3. Combination Treatment of Anti CD38 and IMID in RRMM

The combination with an IMiD and CD38-targeting antibody has improved clinical outcomes in different stages of the disease. The preclinical rationale for this combination is related to the multiple immunomodulatory effects of IMiDs including the increase in NK cell counts and NK cell activity [29]. Lenalidomide (but not bortezomib) as a partner to daratumumab significantly increases ADCC of daratumumab. Enhanced NK cell activity and T cell activity as a synergic effect between IMiDs and daratumumab may also overcome resistance to both agents [30,31,32]. The robust POLLUX trial (NCT02076009) comparing lenalidomide and dexamethasone (Rd) with or without daratumumab until progression or intolerance in patients with relapsed MM after at least one previous line of therapy [33]. The trial included 569 patients with median one previous line of therapy. The median PFS was 44.5 months in Dara-Rd arm and 17.5 months in Rd arm. Dara-Rd reduced risk of progression or death by 56% [34]. The benefit was seen across all patient subgroups including high-risk patients (median 22.6 vs. 10.2 months; hazard ratio (HR), 0.53) [35]. Dara-Rd also showed an unprecedented ORR of 93% in RRMM with 55% of patients reaching complete remission (CR) or better. The minimal residual disease (MRD) negativity rate (10^−5^) was 27% in Dara-Rd arm versus 5% in Rd arm.

Results from the combination of pomalidomide, dexamethasone with or without daratumumab (APOLLO trial, NCT03180736,) and pomalidomide, dexamethasone with or without isatuximab (ICARIA trial, NCT02990338) have been recently reported. There were some differences in eligibility for both trials. For APOLLO, eligible patients had RRMM and received ≥1 prior line of therapy including lenalidomide and a PI, had responded to prior treatment and progressed on or after their last regimen (those with only one prior line of therapy were required to be refractory to lenalidomide). ICARIA recruited patients with ≥2 prior lines of therapy and had not responded to therapy with lenalidomide and a PI alone or in combination. The APOLLO trial included 304 patients with median two prior lines of therapy and 79.6% of patients were refractory to lenalidomide and 68% refractory to PI and 63% to both. Addition of daratumumab improved PFS with median 12.4 vs. 6.9 months (*p* = 0.0018) [36].

The ICARIA study enrolled 307 patients with a median of three prior lines of therapy. All patients were pretreated with lenalidomide and 93% were refractory to lenalidomide and 76% to PI. The median PFS was 11.5 months in the Isa-Pd arm vs. 6.5 months in Pd arm (HR, 0.596, *p* = 0.001) [37]. Subgroup analysis of this trial showed significant improvement of PFS in patients with renal impairment (<60 mL/min/1.73 m^2^), PFS was 9.5 months in Isa-Pd arm and 3.7 months in Pd arm (HR 0.50, *p* not reported) [38]. The effect was also maintained in elderly population including patients >75 years [39].

### 3.4. Combination Treatment of Anti CD38 and PI in RRMM

CD38 antibodies can also be effectively combined with PIs. Combinations with bortezomib as well as carfilzomib have been extensively studied. Combinations with carfilzomib show substantial efficacy in RRMM treatment. Possible mechanisms of synergy include mechanism of immunogenic cell death observed with both agents. [40].

The phase 3 CASTOR study was a randomized trial comparing bortezomib and dexamethasone (Vd) with or without daratumumab in RRMM patients. Vd was discontinued after eight cycles and daratumumab was continued until progression [41]. The median PFS was 16.7 months in Dara-Vd arm compared to 7.1 months in Vd arm (*p* < 0.0001). The ORR was 82.9% in Dara-Vd arm compared to 63.2% in Vd arm (*p* < 0.001) as well as very good partial remission (VGPR), CR rate and MRD negativity rates were significantly better in Dara-Vd arm. Daratumumab arm was also superior in high-risk cytogenetic group (median OFS 11.2 versus 7.2 months; HR, 0.45, *p* = 0.0053) [42]. 

The combination of anti-CD38 and carfilzomib was studied in two very similar large trials, the CANDOR (NCT03158688) and IKEMA (NCT03275285) trial. The CANDOR trial evaluated carfilzomib and dexamethasone (Kd) with or without daratumumab (Dara-Kd). This was a phase 3 open label study that included 466 patients. ORR was 84.3% in Dara-Kd arm versus 74.7% in Kd arm and especially CR rate and MRD negativity (10^−5^) were better in Dara-Kd arm (28.5% and 10.4% CR rate, 17.6% and 3.9% MRD negativity) [43]. The median PFS was 28.6 months in the Dara-Kd group versus 15.2 months in the KD group (HR 0.59) [44].

The results of the IKEMA trial were presented in 2020. This trial evaluated isatuximab, carfilzomib and dexamethasone with or without isatuximab. The study included 302 RRMM patients with 1–3 prior treatment lines. Median PFS was not reached in the Isa-Kd group versus 19.2 months in the Kd group (HR 0.531, *p* = 0.0007), representing a 47% reduction in the risk of disease progression or death for Isa-Kd. The benefit was seen across all subgroups of patients. ORR was 86.6% in Isa-KD arm versus 82.9% in Kd arm. CR rate and MRD negativity (10^−5^) were better in IKd arm (39.7% and 27.6% CR rate, 29.6% and 13.0% MRD negativity). It is important to mention that MRD negativity was reached in 41.4% of patients with VGPR or better in Isa-Kd arm (compared to 22.9% in Kd arm) [45]. An updated analysis showed even better CR rate when isatuximab interference in serum protein electrophoresis was excluded [46]. The trial also included patients with renal impairment with estimated GFR as low as 15 mL/min/1.73m^2^. Analysis of this subgroup of patients showed manageable safety profile as well as higher proportion of patients who recovered renal functions [47]. 

Both trials enrolled a very similar population of patients; an indirect comparison of both trials is summarized in Table 2

### 3.5. Combination Treatment of Anti CD38 in Newly Diagnosed MM (NDMM)

As CD38 is also highly expressed in NDMM patients [48] it is logical step forward to incorporate anti-CD38 MoAbs in frontline therapy. Frontline therapy has been investigated in transplant eligible (TE) as well as transplant ineligible (TI) patients. Bortezomib, melphalan and prednisone (VMP) has long been one of the standard of care regimens for the treatment of elderly myeloma patients [49]. Addition of daratumumab to this combination was studied in the ALCYONE trial (706 patients with median age 71 years). Dara-VMP showed superior results in both PFS and MRD negativity rates in patients treated in daratumumab arm. The ALCYONE trial showed significantly longer PFS in Dara-VMP arm (36.4 months in Dara-VMP and 19.3 months in VMP arm, HR 0.42, *p* < 0.0001). Overall survival benefit was noted as well (HR 0.6, *p* = 0.0003) [50]. ORR was high and was 90.9% in the experimental arm and 73.9% in the control arm. MRD negativity rates (10^−5^) were 22.3% compared to 6.2% in the control arm (*p* < 0.001). Benefit was observed in all subgroups of patients treated in the experimental arm including patients above 75 years of age. High risk cytogenetic group showed PFS improvement with HR 0.78 [51]. 

Another standard of care for elderly patients is the lenalidomide and dexamethasone (Rd) regimen. The MAIA trial included 737 TI patients with NDMM (median age 73 years). Patients were assigned either to lenalidomide plus dexamethasone arm or to daratumumab, lenalidomide and dexamethasone (Dara-Rd) arm. Again, a significant benefit of adding daratumumab to a standard of care regimen was seen. The analysis of MAIA trial with median 47.9 months of follow-up showed that PFS was significantly longer in Dara-Rd arm (median still not reached vs. 34 months; HR, 0.54; *p* < 0.0001). The estimated 48-month PFS rate was 60% with D-Rd vs. 38% with Rd [52]. ORR was high and was 92.9% in Dara-Rd arm and 81.3% in Rd arm. MRD negativity rates (10^−5^) were 24.2% compared to 7.3% in control arm (*p* < 0.001) [53]. Benefit was observed in all subgroups of patients treated in the experimental arm including the patients above 75 years of age. Benefit in high-risk cytogenetic group was moderate however recent meta-analysis showed benefit of daratumumab in NDMM patients [54].

Several other ongoing trials are investigating the addition of anti-CD38 MoAb to a triplet regimen in transplant ineligible patients (see Table 4) for details. This included the phase 3 CEPHEUS and IMROZ studies that evaluate the value of adding daratumumab or isatuximab to VRD.

Daratumumab has also been studied in various clinical trials in newly diagnosed patients eligible for stem cell transplantation. The pivotal CASSIOPEIA study investigated the role of Dara-VTD versus VTD as induction therapy and consolidation treatment post-transplant. Overall, 1085 patients were included (median age 59 years). Patients received four induction cycles of VTD with or without daratumumab followed by autologous stem cell transplantation (ASCT) with melphalan 200 mg/m^2^ conditioning, and then two additional cycles of consolidation treatment with Dara-VTD or VTD. There was a second randomization in patients with PR or better following consolidation, who were scheduled either to daratumumab maintenance or observation. Overall response rate following consolidation was high in both arms (92.2% with daratumumab and 89.9% without). MRD negativity rates (regardless of response) were significantly better in Dara-VTD arm (64% versus 44% in control arm, *p* < 0.0001). Better rate of stringent complete responses was seen across all subgroups of patients except high-risk cytogenetics group. Addition of daratumumab to VTD improved PFS (HR 0.47 (*p* < 0.0001)) which was observed across all subgroups of patients [55]. The analysis of incidence of peripheral neuropathy (PN) showed less PN grade 2 or higher in Dara-VTD arm (33% vs. 38%, HR = 0.73, *p* = 0.004) [56]. Another important trial evaluating the benefit of daratumumab addition to standard VRD was the GRIFFIN trial. This was a phase 2 open label study which enrolled 207 patients eligible for ASCT who received four cycles of VRD +/- daratumumab followed by ASCT with melphalan 200 mg/m^2^ conditioning and two additional cycles of consolidation treatment. The treatment was followed by lenalidomide +/- daratumumab for 2 years or until progression. The treatment potential of both regimens is high with the median PFS not reached in either group. The overall response rate was superior in the daratumumab group (99% for daratumumab arm and 91.8% for control arm, *p* = 0.016). Furthermore, MRD negativity rates were significantly higher in Dara-VRD arm (51.0% versus 20.4% in control arm, *p* < 0.0001). A recently updated analysis confirmed the high efficacy and showed 24-month PFS rates 94.5% and 90.8% for the Dara-VRD and VRD arm, respectively. MRD negativity rates still favored Dara-VRD (62.5% vs. 27.2%, *p* < 0.0001) [57]. Dara-R maintenance therapy is capable of deepening the response over time (stringent CR 63.6% vs. 47.4%) [58].

There are other ongoing phase 2/3 clinical trials incorporating anti-CD38 in upfront treatment. The PERSEUS trial (recruitment completed) incorporated daratumumab to standard VRD as well as to maintenance therapy [59]. The ongoing EMN18 trial compares Dara-VCD versus standard VTD arm and investigates the role of ixazomib +/- daratumumab maintenance treatment. The ongoing EMN24 trial (ISKIA) incorporates isatuximab to carfilzomib, lenalidomide and dexamethasone in both induction and consolidation treatment. Table 3 summarizes the available results of studies with anti-CD38 antibodies and Table 4 summarizes ongoing or planned clinical trial containing anti-CD38.

### 3.6. Toxicity Profiles of Anti-CD38 MoAbs

The CD38-targeting monoclonal antibodies generally represent a safe treatment options for both newly diagnosed as well as relapsed/refractory patients. 

General toxicities on anti-CD38 MoAbs include infusion related reactions, drug induced cytopenias and increased risk of infections. Almost all studies showed similar toxicity profiles when anti-CD38 was added to standard regimens. Table 5 shows a comparison of various toxicities across main clinical trials mentioned in previous paragraphs. 

Infusion related reactions (IRR) are frequent especially during the first dose of MoAb administration. The incidence of all grades IRR can be observed in around 50% of patients during the first dose [14]. Nasal congestion, dry cough, rhinitis, sore throat, and dyspnea are among the most widely reported events. Appropriate preventive measures either pre- or postinfusion help to reduce the incidence of serious adverse reactions. The practical approach of IRR management includes interruption of infusion, administration of additional corticosteroids, antihistamines, and montelukast. The IRRs do not usually develop during subsequent administrations [61]. From clinical perspective, at our institution we were able to safely administer subsequent doses of anti-CD38 even in cases of grade 3-4 IRR during the first infusion. So far, we have not observed any grade 5 IRR toxicity during common practice treatment. 

The relatively long infusion time can also be one of the problems especially in centers experiencing high patient load and especially during the ongoing COVID-19 pandemic (as of year 2020/2021). The first daratumumab infusion is usually administered in 6-h infusion and subsequent doses in 3.5-h infusion. There are however reports showing that 90-min infusion (for 3rd and subsequent doses) is as safe as the standard infusion duration [62,63]. Due to the COVID-19 pandemic, we adopted this strategy and observed no serious reactions during the second and subsequent doses (maintaining standard time of 6 h for the first infusion). Another approach may be an earlier switch to less frequent daratumumab or isatuximab administrations, if this is feasible in terms of disease control, as is suggested in European Myeloma Network recommendations [64]. The other alternative now is subcutaneous administration of daratumumab. The COLUMBA trial showed noninferiority of subcutaneous administration compared to intravenous administration of daratumumab as monotherapy for RRMM patients [65]. Following, PLEIADES study investigated various combination regimens with subcutaneous daratumumab (Dara-VMP, Dara-RD) [66]. In addition, a phase Ib clinical trial is evaluating the safety and tolerability of subcutaneous isatuximab (NCT04045795). An open-label phase I/IIa clinical trial investigating subcutaneous TAK-079 (NCT03439280) is also ongoing [28]. 

There are several reports noting that daratumumab administered prior to stem cell collection can decrease the amount of collected stem cells [67]. About 75% of mobilized CD34+ stem cells also express CD38 at the cell surface, albeit at low densities [68]. Current data have demonstrated a greater requirement for plerixafor use when daratumumab is administered within 1 month prior to stem cell mobilization. The patients also needed more apheresis days to collect the target amount of stem cells. All patients managed to collect adequate stem cell amounts [69].

Respiratory infection rate is believed to be higher when anti-CD38 MoAbs are administered with standard regimens. The long-term treatment has been associated with risk of infections (as in ALCYONE trial, see Table 5 for details). Some studies suggest a higher rate of viral infections in patients treated with daratumumab [70]. The prophylaxis with acyclovir or valaciclovir is generally recommended during the administration of anti-CD38 antibodies [71]. One interesting report by Khan et al. reported high susceptibility to *Listeria* infection which is uncommon under normal circumstances [72]. We have observed one such case of listeria brain abscess during daratumumab treatment at our department as well. Infectious complications may represent underestimated risk [73]. Key issues in prevention are early antibiotic treatment, intravenous immunoglobulin substitution in selected patients and vaccinations. Vaccine response in MM patients is generally impaired compared to healthy controls, but all patients are encouraged to get vaccines against common pathogens (including influenza, *Streptococcus pneumoniae*, and *Haemophilus influenzae*) that represent significant risk in MM. Any administered vaccine should not contain live attenuated virus [74,75]. Although there are currently no data available from prospective trials evaluating COVID-19 vaccine efficacy in MM patients, it is currently recommended to vaccinate all MM patients with any vaccine for COVID-19 because of the potential benefit of preventing severe disease in this vulnerable patient population [76].

One of the practical aspects of anti-CD38 administration is the interference of therapeutic antibodies with response evaluation. Since all MoAbs are complete immunoglobulin molecules (usually IgG class) they can be detected in small amounts by standard immunofixation assays. This fact complicates response assessment especially in IgG myeloma patients where a small gradient can be observed even in patients who are in true complete remission. The International Myeloma Working Group (IMWG) stated that CR is defined as disappearance of original M protein and this problem is only present in patients who have the same isotype as the antibody used. In this case the MoAb band cannot be differentiated from the original M protein band. Usually, the amount of MoAb present in the blood is <2 g/L so interference is only an issue in patients who achieved VGPR or CR. It is possible to use daratumumab specific immunofixation reflex assay (DIRA) to distinguish daratumumab from original M-protein [77]. This assay is based on anti-daratumumab antibody which changes the MoAb migration pattern that could help to distinguish between daratumumab and the patient’s M-protein. Commercial DIRA test is available for daratumumab [78]. Various other assays are being developed for isatuximab since obviously all MoAbs share this ability to interfere. Mass spectrometry has been a successfully used method to distinguish isatuximab from original M-protein [79]. 

Daratumumab causes reactivity in the indirect antiglobulin test via binding to surface CD38 molecule on test red blood cells [80]. Positive indirect antiglobulin test can be observed up to 6 months after daratumumab treatment termination [81]. This fact may lead to delay in compatible blood selection on transfusion departments. One of the possibilities to overcome this problem is to use a neutralization method with an antibody against daratumumab. The Dithiothreitol test (DTT) is the most widely used method. DTT treatment of red blood cells (RBC) can denature or modify certain antigens including CD38 on the surface, allowing DTT-treated RBCs to be used to avoid interference of anti-CD38 antibodies in blood group compatibility tests [82]. However, this method is not routinely available. The best practical approach is generally to phenotype red blood cells of the patient prior to anti-CD38 treatment (or genotype after). At our institution we are currently phenotyping red blood cells in all newly diagnosed MM patients to eliminate this problem during their further therapy. It is necessary to point out that this is a sole laboratory problem—no adverse reactions to blood transfusion have been reported in patients treated with anti-CD38 so far [83].

## 4. Anti SLAMF-7 Monoclonal Antibodies

Signaling Lymphocyte Activation Molecule Family 7 (SLAMF-7) is a consistently expressed glycoprotein on the surface of MM cells. SLAMF7 is also expressed on lymphocytes, especially NK cells, activated T cells, and most B cells [84,85,86]. The SLAM family receptors play important roles in immune regulation. SLAMF-7 works in cooperation with Ewing’s sarcoma-associated transcript 2 (EAT-2). SLAMF-7 together with EAT-2 triggers activating NK cell signals thereby increasing NK cell activity [84,87]. It is important to note that MM cells lack EAT-2 so SLAMF-7 does not provide activation signals. Soluble SLAMF-7 acts as a growth factor for MM cells [88]. Additionally, increase in soluble SLAMF-7 may point to disease progression [89]. Elotuzumab is a humanized monoclonal IgG1 antibody that binds SLAMF7 and is currently approved for treatment of patients with RRMM. Tagging of MM cells and increase in the activity of NK cells is probably the explanation of mechanism of action of elotuzumab. It promotes NK cell dependent ADCC [90]. It lacks other mechanisms of action typical for other MoAbs such as CDC [91]. ADCP activity of elotuzumab has been recently documented in a xenograft MM mouse model [92]. 

### 4.1. Monotherapy

The phase I study evaluating safety of elotuzumab was published in 2012. This study included heavily pretreated patients with RRMM. It included 35 patients with median 4.5 prior lines of therapy. Patients were treated with escalating doses of elotuzumab (0.5–20 mg/kg Q1W). Although the toxicity of the drug was well tolerated, the trial failed to show any clinical meaningful efficacy. Using the EBMT criteria at that time, nine patients were classified as stable disease and the rest experienced progressive disease. Since the tolerability of the drug was good, combination trials with elotuzumab were initiated [93].

### 4.2. Combination Treatment in RRMM

Combination treatment with IMiDs as well as with PIs has been evaluated. An overview of the largest studies is shown in Table 6. Since lenalidomide can enhance NK cell activity, the combination of elotuzumab with lenalidomide and dexamethasone was evaluated in the ELOQUENT-2 trial which led to approval of this combination by FDA and EMA. In fact, elotuzumab was the first MoAb approved for MM patients. In the ELOQUENT-2 trial RRMM patients were treated with Rd +/- elotuzumab until progression or unacceptable toxicity. Eligible patients received one to three previous therapies and had documented disease progression after their most recent therapy. A total 646 patients with median age 66 years and median two prior lines of therapy were included. The median PFS was 19.4 months in Elo-Rd versus 14.9 versus Rd group (HR 0.7, *p* < 0.001). The overall response rate was 79% versus 66% in the control arm. IRRs were reported only in 10% of patients receiving elotuzumab which is a much lower number than what is observed with anti-CD38 MoAbs. There was a higher incidence of herpes zoster in the elotuzumab arm [94]. The final update of this trial was published recently after a minimum follow-up of 70.6 months. The most important observation from this analysis is the prolonged OS in Elo-Rd arm. There was a substantial clinically meaningful benefit of OS; 48.3 months in Elo-Rd compared to 39.6 months in Rd arm (HR 0.82, *p* = 0.0408). Its effect on OS was most prominent in patients above the age of 75 years and in patients with adverse cytogenetics and advanced ISS stage 3 disease [95]. 

The ELOQUENT-3 trial randomized RRMM patients to treatment with pomalidomide, dexamethasone (Pd) with or without elotuzumab until disease progression or death. Eligible patients received two or more previous lines of therapy (including lenalidomide and PI) and their disease was refractory or relapsed and refractory to lenalidomide and a PI. The trial recruited 117 patients with median age 69 years and a median of three prior lines of therapy. Almost all patients were previously exposed to lenalidomide and all patients were exposed to bortezomib. The majority of patients were refractory to lenalidomide (90% in Elo-Pd arm and 84% in Pd arm) and 68% were double refractory to lenalidomide and PI in Elo-Pd arm and 72% in Pd arm. Addition of elotuzumab significantly improved PFS (median PFS: 10.3 months versus 4.7 months; HR 0.54, *p* = 0.008). The PFS benefit was observed across all subgroups of patients including the patients who were refractory to lenalidomide and bortezomib (HR 0.56). The overall response rate was approximately two times higher in the Elo-Pd arm (53% versus 26%). The toxicity profiles were similar in both treatment arms [96]. For the patients with double refractory myeloma these results represent a significant improvement in their outcome and this trial literally put elotuzumab “back into the saddle” since Elo-Pd represents a viable, nontoxic, and manageable option in these cases.

Studies incorporating combinations with bortezomib (i.e., Elo-Vd) were presented with some degree of effect and are summarized in Table 6. Given the fact that bortezomib based regimens are slowly leaving the field of RRMM the use of these regimens did not receive enough attention.

### 4.3. Combination Treatment in NDMM

Elotuzumab was studied in the large phase III trial ELOQUENT-1. Transplant ineligible patients with NDMM were treated with Rd with or without elotuzumab until progression. The trial was conducted as early as 2011 and the results of this trial have never been published. The results are now publicly available through www.clinicaltrials.gov (accessed on 27 March 2021) web page. Just to summarize, the trial did not meet its primary endpoint with no significant benefit on PFS. 

Elotuzumab in combination with other frontline regimens has been studied in several other phase II and III trials (see Table 7 for details). Elotuzumab is also studied in maintenance setting after ASCT [97]. A combination of lenalidomide, bortezomib and dexamethasone with or without elotuzumab as frontline therapy did not show any benefit of adding elotuzumab in patients with high-risk newly diagnosed MM (RVd 33.6 months vs. Elo-RVd 31.5, HR 0,968, *p* = 0·45) [98]. A large German study investigating Elo-RVd versus RVd in transplant eligible MM population also showed no benefit of adding elotuzumab [99].

## 5. Antibody Drug Conjugates

Antibody drug conjugates (ADC) represent an attractive approach to treat various hematologic malignancies [102]. After successful introduction of brentuximab vedotin for the treatment of Hodgkin lymphomas and T cell lymphomas and significant success of revived gemtuzumab ozogamicin in acute myeloid leukemia, myeloma has gained its desired attention with ADCs too [103,104]. The basic idea of these drugs is to deliver the cytotoxic drug (referred to as a payload) directly to the malignant cells. The payload is usually linked to a MoAb via a linker which is noncleavable in the circulation and the release of payload is secured by the degradation of the antibody in lysosome. The antibody is usually internalized via endocytosis and then processed by natural cellular processes leading to cleavage of the linker and release of the payload, and finally killing of the malignant cell. The most challenging issue in ADCs is the selection of target membrane protein [105]. The target should optimally be highly expressed on malignant cells and not be present on other cells to limit the toxicity to normal tissues. It is obvious that such targets are difficult to find. Several targets in MM cells were suggested: BCMA, CD56, CD138, and potentially some others like CD74. The toxins attached to MoAb are usually small cytotoxic molecules seldom used as systemic chemotherapy because of adverse toxic profiles. These drugs (as would be expected from chemotherapeutic agents) cause DNA damage or cell cycle cessation. Calicheamicins or pyrrolobenzodiazepine dimers represent DNA damage mechanism and auristatin derivates (monomethylauristatin F) belong to the group of microtubule inhibitors [106,107].

Out of all possible targets, BCMA has gained the most attention among all other targets. BCMA is beside myeloma cells, only expressed on plasmablasts and mature plasmacytes which makes it an attractive target. BCMA has two known ligands: B-cell activating factor (BAFF) and A Proliferation-Inducing ligand (APRIL). Activation of BCMA leads to activation of NF kappa B pathways creating a prosurvival signal. Results of this process include proliferation, differentiation, and longer survival of plasma cells. Some other mechanisms like interaction with bone marrow environment and osteoclasts have been described [108].

The first-in-class antibody drug conjugate approved by FDA and EMA for MM patients is belantamab mafodotin (GSK28579176). It is an anti-BCMA ADC composed of humanized IgG1 anti-BCMA MoAb conjugated via a noncleavable linker with monomethyl auristatin F (better known as mafodotin). Mafodotin is a potent microtubule inhibitor (blocks tubulin polymerization). Once the drug is internalized and mafodotin is released, it arrests cell cycle in G2/M phase. Its Fc fragment is defucosylated and facilitates other effects typical for MoAbs such as ADCC and ADCP. This also allows to target and kill nondividing MM cells [109]. Belantamab mafodotin was first evaluated in the phase I dose escalation and expansion trial DREAMM-1. This study enrolled 73 heavily pretreated patients with median five prior lines of therapy including 31 (89%) double refractory patients and 13 (37%) patients refractory to daratumumab. Overall response rate was 60%. Two patients reached stringent CR and three additional patients reached CR. The median PFS was 12 months (follow-up 26.5 moths) [110]. Even though the therapy is targeted, off-target effects of ADC administration do occur. The most frequent adverse events are thrombocytopenia reported in 63% of patients and ocular complications (discussed in detail at the end of paragraph) [111]. The ocular, or more specifically corneal events, are experienced in other clinical trials with mafodotin which is likely to be the responsible agent. This study was followed by the DREAMM-2 trial, which led to registration of belantamab mafodotin. This was a two-arm phase II trial. Overall, 95 patients were recruited into the 2.5 mg/kg group and 99 patients into the 3.4 mg/kg group. The drug was administered intravenously every 3 weeks as monotherapy. Patients were heavily pretreated with median seven lines of prior therapy in the 2.5 mg/kg arm and six lines in the 3.4 mg/kg arm. All patients were pretreated with lenalidomide, 98% with bortezomib, 98% with daratumumab. Almost 90% of patients were refractory to lenalidomide (both groups) and 100% to daratumumab in the 2.5 mg/kg arm and 92% in the 3.4 mg/kg arm. Median PFS was 2.9 months in the 2.5 mg/kg arm and 4.9 months in the 3.4 mg/kg arm. A VGPR or better was achieved in 18 (19%) in the 2.5 mg/kg arm and in 20 (20%) of 99 patients in the 3.4 mg/kg arm [112]. The most common toxicities were thrombocytopenia, keratopathy, and IRRs. No grade 4-5 IRRs were observed and overall, 17 patients (18%) experienced grade 1-2 IRR and three patients (3%) grade 3 in the 2.5 mg/kg arm. In total, 15 patients (15%) experienced grade 1-2 IRR and one patient (1%) grade 3 in the 3.4 mg/kg arm. Thrombocytopenia was reported in 33 patients (34%) in the 2.5 mg/kg arm and in 58 patients (59%) in the 3.4 mg/kg arm. Grade 4 thrombocytopenia was reported in 11 (12%) patients in the 2.5 mg/kg arm and in 22 (22%) in the 3.4 mg/kg arm. One patient death was attributed to thrombocytopenia in the 3.4 mg/kg arm [112]. The major concern regarding toxicity of belantamab mafodotin is keratopathy. It is clinically characterized by corneal epithelium changes that lead to blurry vision and dry eye [113]. Transient loss of vision is also possible. Keratopathy was very common; grade 1-2 was seen in 41 patients (43%), grade 3 in 26 patients (27%) in 2.5 mg/kg arm. Even higher rates of keratopathy were observed in the 3.4 mg/kg arm: grade 1-2 in 53 patients (54%), grade 3 in 20 patients (20%) and grade 4 in one patient (1%). Keratopathy was also the main reason for treatment delays and dose reductions (22 patients in the 2.5 mg/kg arm and 27 patients in the 3.4 mg/kg arm) and subsequent discontinuation (one patient in the 2.5 mg/kg arm and three patients in the 3.4 mg/kg arm) [112]. All events were reversible with no permanent loss of vision reported [114]. Prophylactic use of corticosteroid eye drops seems to be ineffective in preventing these events. Dose reduction or dose delays are recommended once corneal events occur. Prophylactic measures include preservative-free artificial tears (4-8 times daily) and eventually cooling eye mask in the first hour of administration of belantamab mafodotin and up to 4 h or as tolerated [112].

Based on the promising efficacy of belantamab mafodotin as single agent, several other studies are now evaluating the combination of belantamab mafodotin with various other agents, their design and results where available can be found in Table 8. Other anti-BCMA ADCs are currently under development and preliminary data have been reported through 2020. The list of these ADCs and corresponding clinical trials with available results can be found in Table 9.

BCMA is not the only possible target for ADCs. Currently ongoing trials evaluating ADCs against other targets are shown in Table 10.

## 6. Conclusions—Beyond the MoAb Therapy

With the introduction of elotuzumab and especially CD38 antibodies, combination regimens including one or more immunotherapeutic agents have become new standards of care for the treatment of RRMM as well as NDMM patients. Therapeutic strategies incorporating MoAbs have especially increased depth of response including many patients achieving MRD negative, which translates into prolonged PFS and OS. This effect observed across all patients’ subgroups, translates into better PFS and, in some already available data, OS as well. The widespread use of MoAbs is mainly given by their common availability and low toxicity profiles. Yet there are still some unanswered questions.

One of the major challenges we are facing is a relapse with disease refractory to CD38 antibody, IMiD, and PI (triple class refractory patients). The MAMMOTH study described the outcomes of triple-refractory patients. The analysis included 275 heavily pretreated patients. The median OS of these patients ranged from 5.6 to 11.2 months based on prior drug exposure and resistance [123]. Addition of an IMiD to daratumumab in daratumumab-refractory patients who were previously refractory to that IMiD, may overcome refractoriness to both drugs [124]. Retreatment of patients refractory to daratumumab with an IMiD or PI-based regimen may show meaningful responses possibly due to immunomodulatory effects of daratumumab [125]. Innovative options are needed for these patients with novel immunotherapeutic approaches. Chimeric antigen receptor T cells (CAR-T) or bi- or trispecific antibodies show promising results in triple-class refractory patients.

Treatment of high-risk patients remains a clinical challenge with still poor outcomes regardless of recent therapeutic advances. Importantly, a large meta-analysis of daratumumab containing regimens (from registration trials) showed clear benefit of adding daratumumab to standard of care regimens in newly diagnosed and relapsed/refractory MM patients with high-risk cytogenetic features [54].

Since MM is a disease of older age, another important population of patients are those above the age of 75 years. These usually frail patients often require dose adjustments leading to inability to deliver appropriate therapy. Analysis of CASTOR and POLLUX trials showed equal benefit of daratumumab containing regimens in a very elderly population and the same was true for isatuximab in ICARIA trial [39,126]. 

Very few data have been reported about the possibility of retreatment with MoAbs, especially anti-CD38. There is one study that reported possibility of retreatment of patients previously treated with daratumumab who were rechallenged with the same molecule. The overall response rate was encouraging although there were very few patients included in this study [127]. There are limited data about possibility of changing anti-CD38 type during subsequent relapses (i.e., daratumumab and isatuximab and vice versa). A small case series of nine patients showed a promising response rate in patients treated with an isatuximab-based regimen after prior daratumumab therapy [128]. There is currently no consensus recommendation about the retreatment with daratumumab [129]. Sequencing of antibodies is another challenging issue. A recent report showed decreased elotuzumab efficacy when used after daratumumab but not vice versa [130]. Prospective studies are needed to address this question. Another challenge represents other less established combinations of various drugs with MoAbs. There is a phase II study ongoing with daratumumab and azacytidine for patients previously treated with daratumumab. Azacytidine is believed to enhance daratumumab response [60]. Preclinical studies strategies investigating resistance to MoAbs have shown potential benefit of combination of daratumumab and all-trans retinoic acid as well as histone deacetylase inhibitors [131,132]. 

MoAbs represent a great therapeutic improvement and in the near future we might be able to treat almost every new patient with an antibody-based regimen upfront to increase the chance of cure and prolong survival.

## Figures and Tables

**Table 1:** Usual dosing of currently approved antibodies.

Drug	Usual Dose	Schedule *	Recommended Premedication
Daratumumab	16 mg/kg i.v. or 1800 mg s.c.	Cycle 1–2 days 1,8,15,22, cycles 3–6 days, cycle 7+ day 1 With DVd or D-VMP other schedule	Dexamethasone, antihistamine, acetaminophen, antileukotriene (montelukast)
Isatuximab	10 mg/kg i.v.	Cycle 1–4 days 1,8,15,22,29, cycle 4+ days 1,15, cycle 18+ day 1	Dexamethasone, antihistamine, acetaminophen
Elotuzumab	10 mg/kg i.v.	Cycle 1–2 days 1, 8, 15, 22, cycle 3+ days 1,15With Elo-Pd other schedule Increase to 20 mg after cycle 2	Dexamethasone, antihistamine, acetaminophen
Belantamab mafodotin	2.5 mg/kg i.v.	Every 3 weeks	None

* schedule may vary according to accompanying regimen.

**Table 2:** Comparison of CANDOR and IKEMA trials.

	CANDOR (NCT03158688) [43]		IKEMA (NCT03275285) [47]	
	Dara-Kd	Kd	Isa-Kd	Kd
Participants	312	154	177	122
Median age (years)	64 (57–70)	64.5 (59–71)	65 (37–86)	63 (33–90)
Median No. of prior therapies	2 (1–2)	2 (1–4)	2 (1–4)	2 (1–4)
HR cytogenetics	15%	17%	23.5%	25.2%
Len refractory	32%	36%	31.8%	34.1%
ORR	84.3%	74.7%	86.6%	82.9%
≥VGPR	69.2%	48.7%	72.6%	56.1%

**Table 3:** Results of the important trials with regimens containing anti-CD38.

Study	Regimen	NCT Number	Phase	Population	No. of Patients	Median PL	R Refractory	V Refractory	≥VGPR	≥CR	MRD-	PFS
Sirius + GEN501 [26]	Daratumumab	NCT01985126, NCT00574288	II	RRMM	148	5	84%	85%	13.5%	4.7%	NA	4.0 m
POLLUX [33]	Dara-Rd vs. Rd	NCT02076009	III	RRMM	286/283	1	3.5 vs. 3.9%	19.9 vs. 16.3	75.8% vs. 44.2%	43.1% vs. 19.2%	22.4% vs. 4.9%	44.5 vs. 17.5 m
CASTOR [41]	Dara-Vd vs. Vd	NCT02136134	III	RRMM	251/247	2	71.3% vs. 80.2 (exposed)	67.3% vs. 69.6 % (exposed)	59.2% vs. 29.1%	19.2% vs. 9%	NA	16.7 vs. 7.1 m
APOLLO [36]	Dara-Pd vs. Pd	NCT03180736	III	RRMM	151/153	2	79.6%	48.0%	51.0% vs. 19.6%	24.5% vs. 3.9%	9% vs. 2%	12.4 vs. 6.9 m
ALCYONE [51]	Dara-VMP vs. VMP	NCT02195479	III	TI NDMM	350/356	0	NAP	NAP	71.1% vs. 49.7%	42.6% vs. 24.4%	22.3% vs. 6.2%	36.4 vs. 19.3 m
MAIA [53]	Dara-Rd vs. Rd	NCT02252172	III	TI NDMM	368/369	0	NAP	NAP	79.3% vs. 53.1%	47.6% vs. 24.9%	24.2% vs. 7.30	NR vs. 34 m
CASSIOPEIA [55]	Dara-VTD VTD	NCT02541383	III	TE NDMM	543/542	0	NAP	NAP	83% vs. 78%	39% vs. 26%	64% vs. 44%	NR vs. NR
GRIFFIN [57]	Dara-VRD vs. VRD	NCT02874742	III	TE NDMM	104/103	0	NAP	NAP	90.9% vs. 73.2%	51.5% vs. 42.3%	51% vs. 20.4%	NR vs. NR
[27]	Isa, Isa-D	NCT01084252	I/II	RRMM	109/55	4	70.6% and 61.8%	65.1% and 67.3%	9.2% and 20%	0%	0%	4.9 and 10.2 m
ICARIA [37]	Isa-Pd vs. Pd	NCT02990338	III	RRMM	154/153	3	94.0% vs. 92.0%	77% vs. 75%	32.0% vs. 9.0%	5% vs. 1%	5% vs. 0%	11.5 vs. 6.5 m

Abbreviations: Dara: daratumumab, Isa: isatuximab, V: bortezomib, R: lenalidomide, P: pomalidomide, D/d: dexamethasone, ORR: overall response rate, VGPR: very good partial remission, CR: complete remission, PFS: progression free survival, MRD: minimal residual disease, m: month.

**Table 4:** Selection of ongoing trials with regimens containing anti-CD38.

Study	NCT Number	Phase	Regimen	Population	Enrollment Estimate	Status
PERSEUS [59]	NCT03710603	III	Dara-VRD + ASCT + Dara-VRD consolidation + Dara-R maintenance vs. VRD + ASCT + VRD consolidation + R maintenance	TE NDMM	690	Recruitment completed
EMN18	NCT03896737	II	Dara-VCD + 1-2x ASCT + Dara-VCD consolidation vs. VTD + 1-2x ASCT + VTD consolidation + 2nd R maintenance Ixa vs. Dara-Ixa	TE NDMM	400	Recruiting
EMN24 (ISKIA)	NCT04483739	III	Isa-KRD + ASCT + Isa-KRD consolidation vs. KRD + ASCT + KRD consolidation	TE NDMM	300	Recruiting
	NCT02513186	I	Isa-VCD and Isa-VRd	TI NDMM	88	Recruitment completed
IMROZ	NCT03319667	III	Isa-VRd vs. VRd	TI NDMM	475	Recruitment completed
	NCT04083898	I/II	Isa-Bendamustin-Prednisone	RRMM	37	Recruiting
	NCT03194867	I/II	Isa-celiplimab	RRMM	109	Recruitment completed
	NCT04240054	II	Isa-VCD	TE NDMM	41	Not yet recruiting
GMMG HD7	NCT03617731	III	Isa-VRD induction + R vs. Isa-R maintenance	TE NDMM	662	Not yet recruiting
LIGHTHOUSE	NCT04649060	III	Dara-melflufen	RRMM	240	Recruiting
CONFIRM	NCT03836014	III	Dara-R continuous vs. fixed 24 m duration	NDMM	434	Recruiting
DARAZADEX [60]	NCT04407442	II	Dara-Azacytidine	RRMM	23	Recruiting

Abbreviations: Dara: daratumumab, Isa: isatuximab, V: bortezomib, R: lenalidomide, P: pomalidomide, K: carfilzomib, D/d: dexamethasone, ASCT: autologous stem cell transplantation, TE: transplant eligible, TI: transplant ineligible, NDMM: newly diagnosed multiple myeloma, RRMM: relapsed/refractory multiple myeloma.

**Table 5:** The most common toxicities of anti-CD38 containing regimens.

Study	IRR (Any Grade)	Thrombocytopenia (Grade 3 + 4)	Neutropenia (Grade 3 + 4)	Infection (Grade 3 + 4)	Pneumonia (Grade 3 + 4)
POLLUX (Dara-Rd, NDMM)	47.7%	12.7%	51.9%	28.3%	7.8%
CASTOR (Dara-Vd, RRMM)	45.3%	45.3%	12.8%	21.4%	8.2%
APOLLO (Dara-Pd, RRMM)	6% (s.c.)	NR	68.0%	NR	13.0%
ALCYONE (Dara-VMP, NDMM)	27.7%	34.4%	39.9%	23.1%	11.3%
MAIA (Dara-Rd, NDMM)	40.9%	NR	50.0%	32.1%	13.7%
CASSIOPEIA (Dara-VTD, NDMM)	35.0%	11.0%	28.0%	22.0%	4.0%
GRIFFIN (Dara-VRd, NDMM)	42.4%	16.2%	41.4%	23.2%	8.1%
ICARIA (Isa-Pd, RRMM)	38.0%	16.0%	61.0%	NR	16.0%
CANDOR (Dara-Kd, RRMM)	40.9%	24.0%	9.0%	29.0%	12.0%
IKEMA (Isa-Kd, RRMM)	44.6%	19.2%	23.8%	NR	32.2%

Abbreviations: Dara: daratumumab, Isa: isatuximab, V: bortezomib, R: lenalidomide, P: pomalidomide, K: carfilzomib, D/d: dexamethasone, IRR: infusion related reaction, NR: not reported, s.c.: subcutaneous.

**Table 6:** Results of the important trials with regimens containing elotuzumab.

Study Regimen	NCT Number	Phase	Population	No. of Patients	m PL	Len Refractory	Bort Refractory	IRR % (Any Grade)	≥VGPR	≥CR	PFS
Elotuzumab [93]	NCT00425347	I	RRMM	35	5	82.4% exposed	82.4% exposed	58.8%	0	0	NA
Elo-Rd vs. Rd (ELOQUENT-2) [94]	NCT01239797	III	RRMM	321/325	2	0%	22% vs. 21%	10%	33% vs. 28%	4% vs. 7%	19.4 vs. 14.9 m
Elo-Pd vs. Pd (ELOQUENT-3) [96]	NCT02654132	II	RRMM	60/57	3	90% vs. 84%	78% vs. 82%	5%	20% vs. 9%	8% vs. 2%	10.3 vs. 4.7 m
Elo-Vd vs. Vd [100]	NCT01478048	II	RRMM	77/75	1-3	NR	0%	5%	37% vs. 27%	4% vs. 4%	9.7 vs. 6.9 m

Abbreviations: Elo: elotuzumab, V: bortezomib, R: lenalidomide, P: pomalidomide, d: dexamethasone, RRMM: relapsed/refractory multiple myeloma, NR: not reached, IRR: infusion related reaction, VGPR: very good partial remission, CR: complete remission, PFS: progression free survival.

**Table 7:** Selected ongoing trials with regimens containing elotuzumab.

Study Regimen	NCT Number	Phase	Population	Enrollment Estimate	Status
Elo-Pd + 2nd ASCT	NCT03030261	II	RRMM	40	Recruiting
Elo-KRd	NCT02969837	II	NDMM	55	Recruiting
Elo-Rd + ASCT	NCT02843074	II	TE NDMM	55	Completed
Elo-VRd vs. VRd [98]	NCT01668719	II	TI NDMM	100	Completed
Elo-VRd + ASCT (GMMG-HD6) [101]	NCT02495922	III	TE NDMM	564	Completed

Abbreviations: Elo: elotuzumab, V: bortezomib, R: lenalidomide, P: pomalidomide, d: dexamethasone, RRMM: relapsed/refractory multiple myeloma, ASCT: autologous stem cell transplantation, TE: transplant eligible, NDMM: newly diagnosed multiple myeloma, RRMM: relapsed/refractory multiple myeloma.

**Table 8:** Ongoing selected trials with belantamab mafodotin.

Name	Phase		NCT Number	Target Patients	Population
DREAMM-3	III	Belantamab mafodotin + Pd	NCT04162210	380	RRMM
DREAMM-4	I/II	Belantamab mafodotin 2.5/3.4 mg/kg+ pembrolizumab	NCT03848845	40	RRMM
DREAMM-5	I/II	Belantamab mafodotin	NCT04126200	464	RRMM
DREAMM-6	II	Belantamab mafodotin + Rd of +Vd	NCT04246047	123	RRMM
DREAMM-7	III	Belantamab mafodotin + Vd vs. Dara-Vd	NCT04246047	478	RRMM
DREAMM-8	III	Belantamab mafodotin + Pd vs. PVd	NCT04484623	450	RRMM
DREAMM-9	III	VRD +/- belantamab mafodotin	NCT04091126	810	TI NDMM
DREAMM-12	I	Belantamab mafodotin safety in renal impairment	NCT04398680	40	RRMM
DREAMM-13	I	Belantamab mafodotin safety in hepatic impariment	NCT04177823	40	RRMM

Abbreviations: dara: daratumumab, V: bortezomib, R: lenalidomide, P: pomalidomide, d: dexamethasone, RRMM: relapsed/refractory multiple myeloma, TI: transplant ineligible, NDMM: newly diagnosed multiple myeloma, RRMM: relapsed/refractory multiple myeloma.

**Table 9:** Results of selected trials with ADCs.

Drug	NCT Number	Phase	Target	Payload	Mechanism of Action	No. of Patients	Prior Lines	ORR	Major Toxicities
Belantamab mafodotin (DREAMM-1) [110]	NCT02064387	I	BCMA	MMAF	Tubulin inhibitor	35	5	60%	Thrombocytopenia, corneal events
Belantamab mafodotin (DREAMM-2) [112]	NCT03525678	II	BCMA	MMAF	Tubulin inhibitor	97/99	6/7	31%/ 35%	Thrombocytopenia, corneal events
AMG224 [115]	NCT02561962	I	BCMA	Mertansine	Tubulin inhibitor	29/11	7	21%/ 27%	Thrombocytopenia, fatigue, musculoskeletal pain, myalgia
MEDI2228 [116]	NCT03489525	I	BCMA	Pyrrolobenzodiazepine dimer	DNA damage	82	2–11 lines	61.0%	Photophobia, thrombocytopenia, rash
Indatuximab-ravtansine [117]	NCT01001442	I	CD138	DM4	Tubulin inhibitor	35	7	6%	Diarrhea, fatigue, nausea
Indatuximab-ravtansine+Rd or+Poma-dex [118]	NCT01638936	I	CD138	DM4	Tubulin inhibitor	64	1–6 lines	77%/ 79%	Diarrhea, fatigue, and nausea
Lorvotuzumab-mertansine [119]	NCT00991562	I	CD56	DM1	Tubulin inhibitor	37	1–6 lines	6%	Neuropathy

Abbreviations: MMAF: monomethyl auristatin F, DM1: mertansine, DM4: ravtansine, ORR: overall response rate.

**Table 10:** Ongoing/planned trials for other ADCs.

Drug	NCT Number	Phase	Target	Payload	Mechanism of Action
CC-99712	NCT04036461	I	BCMA	Maytansinoid	Microtubule inhibitor
TAK-169 [120]	NCT04017130	I	CD38	Shiga-like toxin A subunit	Ribosome inactivation
TAK-573 [121]	NCT03215030	I	CD38	Attenuated interferon-α	Direct antiproliferative
STRO-001	NCT03424603	I	CD74	Maytansinoid	Microtubule inhibitor
HDP-101 [122]	preclinical	I	BCMA	Amanitin	RNA polymerase II inhibitor

## Data Availability

Not applicable.

## Abbreviations

ADC	antibody drug conjugate
ADCC	antibody dependent cellular cytotoxicity,
ADCP	antibody dependent cellular phagocytosis
APC	antigen presenting cell
ASCT	autologous stem cell transplantation
BCMA	B cell maturation antigen
CDC	complement dependent cytotoxicity
CR	complete remission
d/D	dexamethasone
Dara	daratumumab
DIRA	daratumumab specific immunofixation reflex assay
DM1	mertansine
DM4	ravtansine
EAT-2	Ewing’s sarcoma-associated transcript 2
Elo	elotuzumab
EMA	European Medicines Agency
FDA	Food and Drug Administration
HR	hazard ratio
IMiD	immunomodulatory drug
IMWG	International Myeloma Working Group
IRR	infusion related reaction
Isa	isatuximab
K	carfilzomib
m	month
MMAF	monomethyl auristatin F
MoAb	monoclonal antibody
MRD	minimal residual disease
NDMM	newly diagnosed multiple myeloma
NK	natural killer
NR	not reached
ORR	overall response rate
P	pomalidomide
PI	proteasome inhibitor
PFS	progression free survival
PN	peripheral neuropathy
R	lenalidomide
RBC	red blood cell
RRMM	relapsed/refractory multiple myeloma
SLAMF-7	Signaling Lymphocyte Activation Molecule Family 7
TE	transplant eligible
TE	transplant eligible
V	bortezomib
VGPR	very good partial remission
